# Structural analysis of fungus-derived FAD glucose dehydrogenase

**DOI:** 10.1038/srep13498

**Published:** 2015-08-27

**Authors:** Hiromi Yoshida, Genki Sakai, Kazushige Mori, Katsuhiro Kojima, Shigehiro Kamitori, Koji Sode

**Affiliations:** 1 Life Science Research Center and Faculty of Medicine, 1750-1, Ikenobe, Miki-cho, Kita-gun, Kagawa University, Kagawa 761-0793, Japan; 2 Department of Biotechnology and Life Science, Graduate School of Engineering, Tokyo University of Agriculture and Technology, 2-24-16, Naka-cho, Koganei, Tokyo 184-8588, Japan; 3 Ultizyme International Ltd., 1-13-16, Minami, Meguro, Tokyo 152-0013, Japan

## Abstract

We report the first three-dimensional structure of fungus-derived glucose dehydrogenase using flavin adenine dinucleotide (FAD) as the cofactor. This is currently the most advanced and popular enzyme used in glucose sensor strips manufactured for glycemic control by diabetic patients. We prepared recombinant nonglycosylated FAD-dependent glucose dehydrogenase (FADGDH) derived from *Aspergillus flavus* (AfGDH) and obtained the X-ray structures of the binary complex of enzyme and reduced FAD at a resolution of 1.78 Å and the ternary complex with reduced FAD and D-glucono-1,5-lactone (LGC) at a resolution of 1.57 Å. The overall structure is similar to that of fungal glucose oxidases (GOxs) reported till date. The ternary complex with reduced FAD and LGC revealed the residues recognizing the substrate. His505 and His548 were subjected for site-directed mutagenesis studies, and these two residues were revealed to form the catalytic pair, as those conserved in GOxs. The absence of residues that recognize the sixth hydroxyl group of the glucose of AfGDH, and the presence of significant cavity around the active site may account for this enzyme activity toward xylose. The structural information will contribute to the further engineering of FADGDH for use in more reliable and economical biosensing technology for diabetes management.

The flavin adenine dinucleotide (FAD)-dependent glucose dehydrogenases (FADGDHs; EC 1.1.5.9) comprise oxidoreductases that catalyze the oxidation of first hydroxyl group of glucose and other sugar molecules, using FAD as the primary electron acceptor (systematic name; D-glucose:acceptor 1-oxidoreductase). FADGDHs use a variety of external electron acceptors (but not oxygen). The reported FADGDHs can be divided into three distinct groups according to their origin^1^. FADGDHs have been found as extracellular enzymes in fungi, as membrane-binding protein of Gram-negative bacteria, and as cytosolic enzymes in some insects. Recently, fungus-derived FADGDH has received attention as the enzyme for glucose monitoring, especially self-monitoring of blood glucose (SMBG), exploiting its oxygen insensitivity and narrow substrate specificity, in particular its lack of activity toward maltose.

Since the first article on electrochemical glucose sensing^2^, glucose oxidase (GOx) (β-D-glucose:oxygen 1-oxidoreductase, EC 1.1.3.4) has been used as the gold standard enzyme for blood glucose monitoring. GOx is a flavoprotein that catalyzes the oxidation of β-D-glucose at its first hydroxyl group, using molecular oxygen as the electron acceptor, to produce D-glucono-delta-lactone and hydrogen peroxide. GOx is a homodimeric enzyme with an FAD molecule noncovalently but tightly bound at the active site of each 80-kDa subunit (as a glycosilated enzyme). GOx is a representative enzyme of the glucose/methanol/choline (GMC) oxidoreductase family. Because GOx inherently reacts with oxygen, glucose oxidoreductases that do not use oxygen, glucose dehydrogenases, have become the major enzyme for glucose sensing employing artificial electron acceptors (also referred to as electron mediators or redox dyes). Among them, glucose dehydrogenase using pyrroloquinoline quinone (PQQ) as the cofactor (PQQGDH) has become the major enzyme after GOx, in view of its high catalytic efficiency.

However, because of the broad substrate specificity of PQQGDH, which can lead to potentially fatal errors in glucose sensing^3^, fungus-derived FADGDH is now becoming the most popular and reliable enzyme for glucose sensing. Fungus-derived FADGDH was first reported from *Aspergillus oryzae* in 1937^4^ and characterized decades later^5^,^6^,^7^,^8^,^9^. FADGDHs from other fungi, *A. niger*^10^ and *A. terreus*^11^, have since been studied. We previously reported the screening of a fungal genome database using GOx active-site sequence motifs^12^. Recently, several recombinant fungus-derived FADGDHs have been prepared and studied, including enzymes from *A. terreus*^13^, *Glomerella cingulate*^14^,^15^, and *Pycnoporus cinnabarinus*^16^. With increased demands for more accurate glucose sensing systems that meet the requirements of ISO15197-2013^17^ and FDA guidelines^18^, further engineering is still required to improve the enzymatic properties of FADGDH, including stability and substrate specificity^19^. We recently reported the improvement of the thermal stability of FADGDH by introduction of a disulfide bond into the molecule, comparing the GOx three-dimensional (3D) structure with a model structure of FADGDH^20^. However, despite the long history of fungus-derived FADGDHs, no 3D structural information is currently available. Such a structure is required for further structure-based, state-of-the-art biomolecular engineering studies of this industrially significant enzyme.

Here we report the 3D structure of fungus-derived FADGDH. We obtained the X-ray structures of binary complex of enzyme and reduced FAD at a resolution of 1.78 Å, and the ternary complex with reduced FAD and D-glucono-1,5-lactone (LGC) at a resolution of 1.57 Å. These structures revealed the overall structure similarity with GOx and also the active site structure with residues responsible for FAD and substrate binding. We discuss the detailed structural differences and similarities between FADGDH and GOx, including residues responsible for the difference between a dehydrogenase and an oxidase.

## Results

### Overall structure

**Figure 1:**
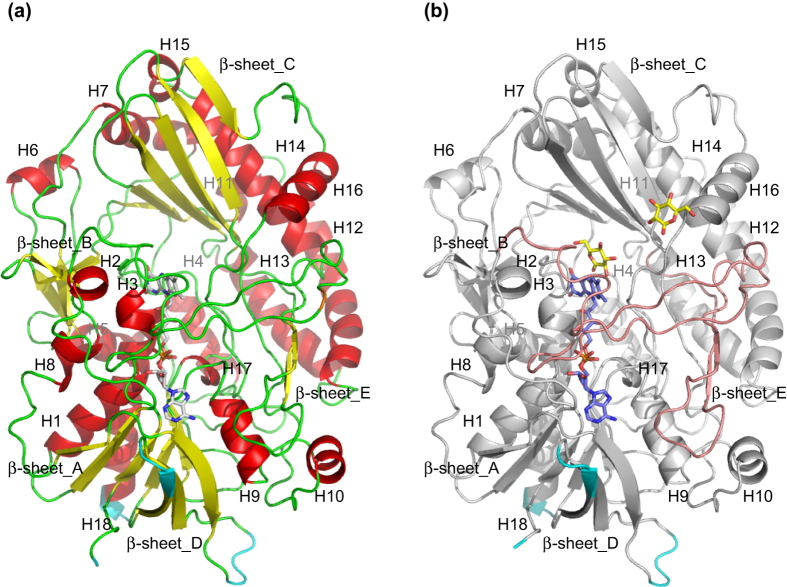
Overall structures of AfGDH alone and AfGDH in complex with D-glucono-1,5-lactone. (**a**) AfGDH. (**b**) AfGDH/LGC (protein, silver; FAD, purple; LGC, yellow). Two long loops cover the FAD molecule (pink). Regions with low electron densities are indicated in cyan.

**Figure 2:**
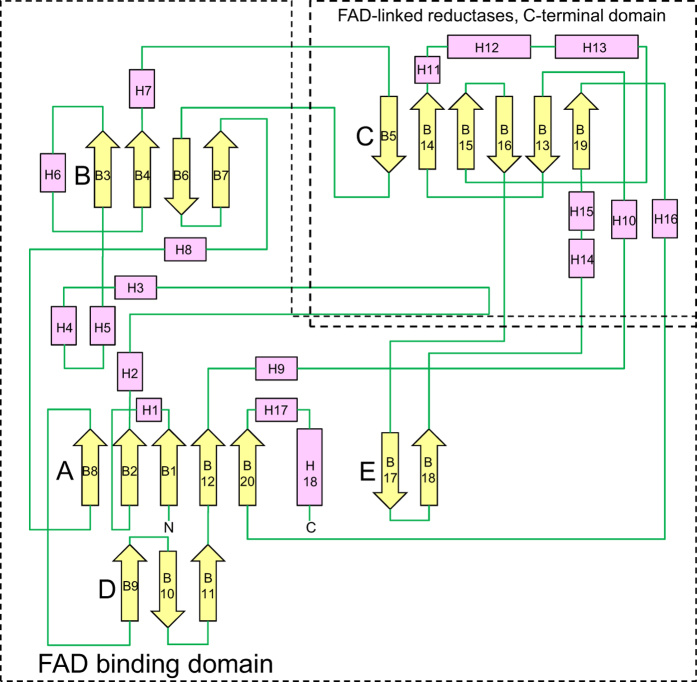
Topology model of AfGDH. β strands and helices are represented by arrows and rectangles, respectively.

The overall structures of *Aspergillus flavus* glucose dehydrogenase (AfGDH) and AfGDH in complex with LGC (AfGDH/LGC) are shown in Fig. 1(a,b), respectively. Both AfGDHs (18 helices, 20 β strands) consist of two major domains. The FAD-binding domain includes a three-layer (β_3_β_5_α_3_) [(B9, B10, B11,), (B8, B2, B1, B12, B20,), (H1, H5, H18)] sandwich structure with short eight α helices (H2, H3, H4, H6, H7, H8, H9, H17) and an irregular β sheet (B3, B4, B6, B7), and a long loop containing a short antiparallel β sheet (B17, B18). The C-terminal domain contains a large six-stranded antiparallel β sheet (B5, B14, B15, B16, B13, B19) surrounded by six α helices (H11, H12, H13, H14, H15, H16) and an additional short α helix (H10) (Fig. 2).

As solved by molecular replacement using the structure of *A. niger* glucose oxidase (AnGOx) [Protein Data Bank (PDB) ID: 1CF3^21^], the whole structure of AfGDH is similar to that of the fungal GOxs AnGOx (1CF3, 1GAL^22^, 3QVP^23^) reported till date, with r.m.s.d. 1.7 Å and 35% identity, and *Penicillium amagasakiense* glucose oxidase (PaGOx, 1GPE^21^) with r.m.s.d. 1.7 Å and 34% identity by Dali search^24^. The structural topology of AfGDH is similar to that of GOxs^21^,^22^.

Low or invisible electron densities were found in the residues at the N terminus (1-3), the surface loop region (243–246) between B9 and B10, the β turn (259–261) between B10 and B11, and the C terminus (569–571) owing to their flexibility or highly disordered nature. These regions are indicated in cyan in Fig. 1.

### FAD-binding site

**Figure 3:**
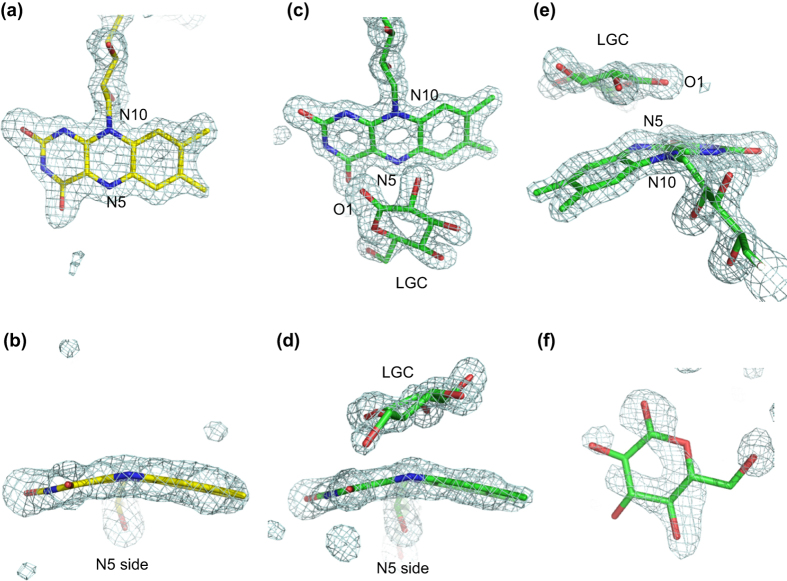
SA-omit maps of the bent isoalloxazine ring of FAD and the bound gluconolactone. The bent isoalloxazine ring of FAD observed in AfGDH with SA-omit maps contoured at 3.0 σ (**a**,**b**). The bound gluconolactone and the bent isoalloxazine ring of FAD in AfGDH/LGC with SA-omit maps contoured at 4.0 σ (**c**–**e**). The bound LGC on the protein surface with SA-omit maps contoured at 3.0 σ (**f**).

In the FAD-binding site of AfGDH, the FAD molecule occupies a narrow channel, as has been reported for GOxs (1GAL, 1CF3). The long loop between H2 and H3, including the potential glycosylation site Asn69, and another long loop containing a two-stranded antiparallel β sheet (B17 and B18) between B16 and H14 cross and cover the FAD molecule like lids. The bound FAD molecule was refined as a reduced FAD cofactor (FADH_2_), observed not only in AfGDH/LGC but also in AfGDH alone (Fig. 3). Both FAD molecules showed clear electron density maps for bending of the isoalloxazine ring of reduced FAD. The conformations of this ring in FAD in the redox states have been reported in the structure of FAD-containing NADH-dependent ferredoxin reductase^25^. The isoalloxazine ring of the oxidized FAD shows a planar conformation, but the ring of reduced FAD in the hydroquinone or semiquinone state is bent at the N5 and N10 of FAD. This finding suggests that both N5 and N10 of FAD are in an *sp*^3^ configuration in the reduced state, and that the N5 of FAD changes to an *sp*^2^ configuration with the remaining *sp*^3^ configuration of the N10 of FAD in the semiquinone state. In the structure of AfGDH alone, we could not identify the bent isoalloxazine ring conformation showing the hydroquinone or semiquinone state, but it clearly shows a bend, as observed in AfGDH/LGC, and was refined as reduced FAD.

Selected hydrogen bond and van der Waals contact distances to the FAD cofactor on AfGDH and AfGDH with bound LGC are presented in Supplementary Table S1. Both FADs showed similar interaction with enzymes, but there was an intriguing difference around O2F, N3F, and O4F of the isoalloxazine ring surrounding Gly94-Met95-Ala96 at the *Re* face of the carbonyl in LGC. The interactions between the isoalloxazine ring and the main chain of Gly94-Met95-Ala96 were diminished in AfGDH/LGC. The Cα-C of Gly94 was rotated and the positions of the N and O of Gly94 changed, resulting in Gly94 interaction only with O4F (Supplementary Figure S1).

### Structure of the substrate-binding site

AfGDH/LGC contains two LGC molecules, which were found in the catalytic site and at the protein surface. The electron density of the LGC bound on the surface of protein was relatively weak (Fig. 3 (f)). The bound LGC was located at inter-molecular surface in the crystal, forming hydrogen bonds with Lys76, Asn499, (Asn346), and two water molecules (Supplementary Figure S2). The label with parenthesis belongs to the neighboring molecule in molecular packing. Therefore, we focused on the LGC bound in the catalytic site in this study.

**Figure 4:**
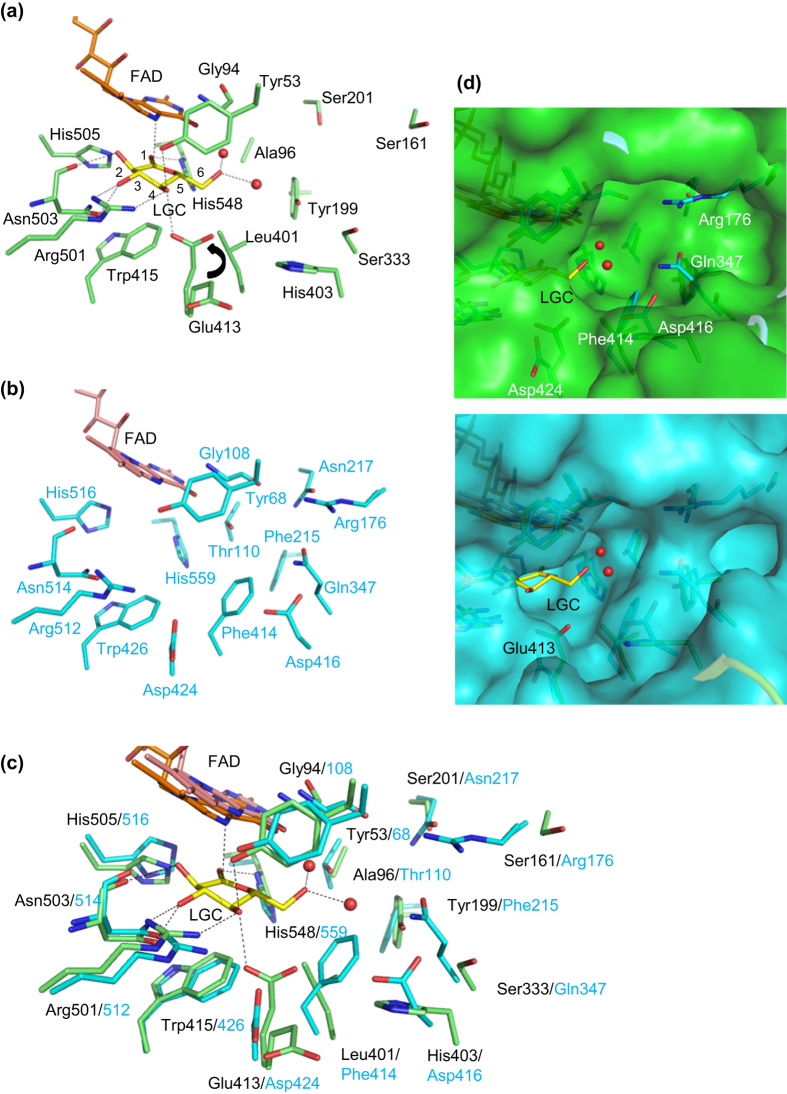
Comparison of active site structures between AfGDH/LGC and AnGOx (1CF3). Active site structures of (**a**) AfGDH/LGC (protein, green; FAD, orange; LGC, yellow) and (**b**) AnGOx (protein, cyan; FAD, pink). (**c**) Comparison of active site structures between AfGDH/LGC and AnGOX. The active site structure of (**b**) was superimposed onto (**a**). (**d**) Representation of the surface of AfGDH/LGC (top) and AnGOx (bottom) on (**c**). Labels of nonconserved residues are represented by AfGDH/LGC and followed by AnGOx. Water molecules interacting with LGC are represented as red spheres.

The active site structure of AfGDH/LGC is compared with that of AnGOx in Fig. 4. Selected hydrogen bond and van der Waals contact distances to LGC are presented in Supplementary Table S2. The residues interacting with LGC (Tyr53, Arg501, Asn503, His505, His548) were conserved in AnGOx (also in PaGOx). Only one residue, Glu413, was not conserved, and it corresponds to Asp424 in AnGOx (Asp428 in PaGOx). In AfGDH/LGC, the side chain of Glu413 causes reorientation and interacts with LGC [Fig. 4(a)]. In addition, Leu401 and Trp415 provide a hydrophobic environment around the bound LGC on the *Si* face, although the indole ring of Trp415 is unlikely to engage in a CH-π interaction with the pyranose ring of LGC. In the vicinity of the active site of AnGOx, Arg176 approaches the substrate-binding site and is located at the position forming hydrogen bonds with Asn217 and Gly108 and has van der Waals contact with Gln347 (these residues are conserved in PaGOx, Arg180, Asn221, Gly112, and Gln351). Unlike AnGOx, the Arg176, Asn217, and Gln347 of AnGOx correspond to the Ser161, Ser201, and Ser333 of AfGDH. Thus, the Ser161 of AfGDH corresponding to the Arg176 of AnGOx (Arg180 of PaGOx) belonging to the loop between B4 and H7 does not approach the active site, and a cavity is present in the vicinity of the substrate-binding site, whereas the conserved Arg and Gln of GOxs occupy a part of this cavity (Fig. 4).

### Site-directed mutagenesis studies on His505 and His548

Among the residues interacting with LGC, His505 and His548 were subjected for Ala substitution. These residues were conserved in both AnGOx(His516/His559) and in PaGOx(His520/His563), which were reported as a catalytic pair in GOxs. His505Ala and His548Ala mutants were prepared by site-directed mutagenesis, and purified enzymes were biochemically characterized, by comparing wild type enzyme. Both mutant enzymes showed drastic decrease in the enzymatic activity (His505Ala; (5.16 ± 0.50) × 10^−5^ U/mg, His548Ala; (1.98 ± 0.02) × 10^−3^ U/mg ) compared with wild type (207.6 ± 6.5 U/mg). Both mutant enzymes showed characteristic two peaks (380 nm and 460 nm) for oxidized FAD, as wild type enzyme (Supplementary Figure S3). The addition of glucose to the wild type enzyme resulted in the disappearance of the peaks showing the formation of reduced FAD. The addition of glucose to His548Ala also resulted in the disappearance of characteristic spectrum of oxidized FAD after a period of incubation. However, His505Ala did not change this peak after the addition of glucose. These observations indicated that both His505 and His548 have crucial role in the catalytic activity, as same as those observed in the conserved His residues in GOxs.

## Discussion

We report the first 3D structure of fungus-derived glucose dehydrogenase using FAD as a cofactor in complex with LGC.

There are two more distinct groups of FADGDHs according to their origin. FADGDHs have been found as cytosolic enzymes in some insects. FADGDH from insect *Drosophila melanogaster* was reported^26^,^27^,^28^,^29^, however the information is limited within its potential physiological role. Its enzymatic characterization and recombinant production have yet been remained to be reported. FADGDH was also reported from Gram-negative bacteria^30^. Bacterial FADGDH is a hetero-oligomeric enzyme complex made up of a catalytic subunit harboring FAD in its redox center, a multiheme cytochrome-complex electron-transfer subunit, and a chaperone-like subunit required for proper folding and secretion of the catalytic subunit^31^,^32^,^33^,^34^. Bacterial FADGDHs have unique direct electron transfer ability with electrode^35^,^36^. The protein engineering studies of the active site in catalytic subunit have been also reported to eliminate the enzyme activity toward disaccharides^37^,^38^. However, none of these FADGDHs’ structures have been elucidated. The elucidation of fungal FADGDH structure may advance the understanding of the structure and function of other FADGDHs.

The subunit structure of AfGDH is similar to that of AnGOx and PaGOx, but the recombinant AfGDH is unlikely to form a dimer conformation, according to PISA^39^. In fact, size exclusion chromatography analysis of AfGDH revealed that recombinantly prepared nonglycosylated AfGDH eluted at an elution volume corresponding to its monomer, unlike GOx, which is eluted at an elution volume corresponding to its dimer (Supplementary Figure S4). The reported structures of AnGOx (1GAL, 1CF3, 3QVP) and PaGOx (1GPE) show a dimer form and contain extended carbohydrate residues indicating glycosylation at the structurally identical position, namely one of the potential *N*-glycosylation sites in the long loop between the H4 and H5 of both GOxs (the Asn89 of AnGOx and Asn93 of PaGOx). The extended carbohydrates contribute to stabilizing the dimer form by linking dimers with hydrogen bonds to form closed dimers (Supplementary Figure S5 (a)). In AfGDH, the corresponding residue is not Asn but has a potential glycosylation site, Asn69, in the corresponding long loop between the H2 and H3 of AfGDH (Supplementary Figures S5, S6). AnGOx and PaGOx have eight and seven potential glycosylation sites, respectively, and *N*-acetylglucosamine molecules were found at four conserved sites in both structures. Given that the recombinant AfGDH also has 10 potential glycosylation sites (N-X-S/T), including Asn69, as suggested by NetNGlyc 1.0 (http://www.cbs.dtu.dk/services/NetNGlyc/), native AfGDH is expected to be glycosylated as AnGOx and PaGOx.

The region of the β turn between B10 and B11, including highly disordered residues (259–261) of AfGDH, is directed toward the opposite side of the corresponding β turn of GOxs, which is located at the dimer interface and contributes to the dimer conformation of GOxs. The β turn between B10 and B11 of AfGDH is directed toward the N-terminal region containing a short α helix of GOxs and is located between the N-terminal region and the corresponding β turn of GOxs. The observed position of the β turn in recombinant AfGDH is expected to prevent dimer formation due to the steric hindrance at the most plausible dimer interface (Supplementary Figure S5 (b)).

**Figure 5:**
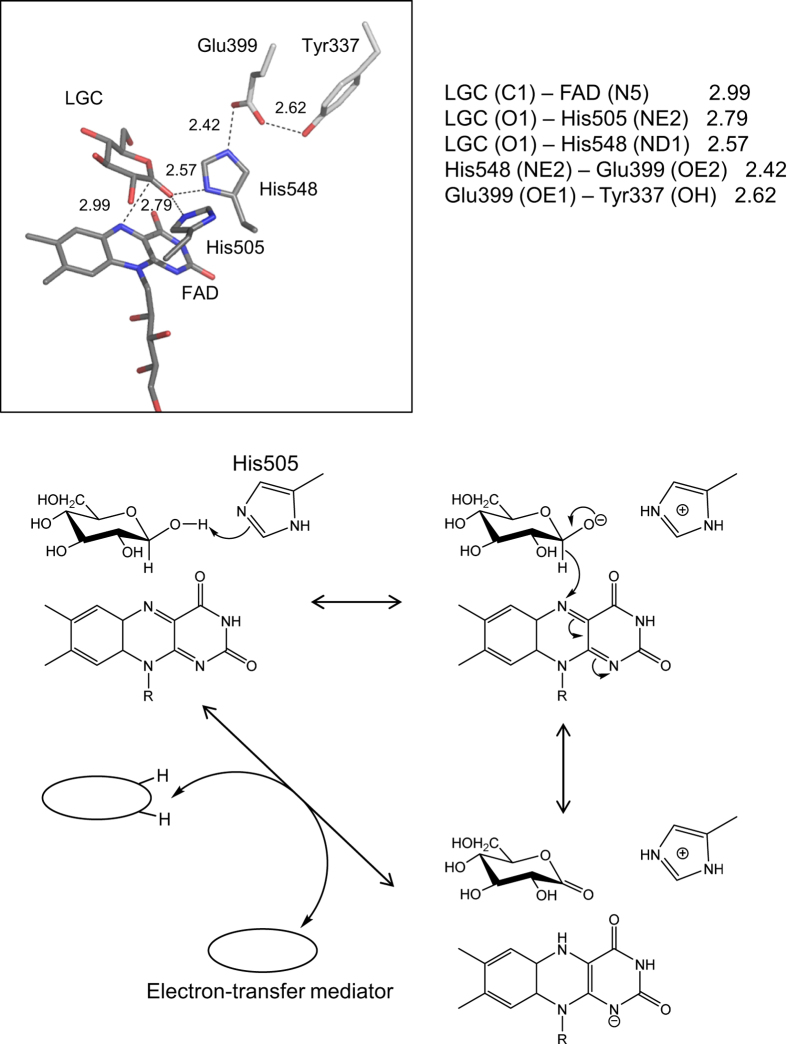
Interactions between catalytic site and LGC with hydrogen-bond distances, and putative reaction mechanism of AfGDH.

The residues responsible for the catalytic reaction have previously been reported in a variety of the GMC oxidoreductases. In GOx^21^,^22^, cholesterol oxidase (ChOx)^40^, choline oxidase (CO)^41^, aryl–alcohol oxidase (AAO)^42^, pyranose 2-oxidase (P2O)^43^, pyranose dehydrogenase (PDH)^44^ and cellobiose dehydrogenase (CDH)^45^, a His/His or His/Asn pair is expected to function as a catalytic pair on the basis of crystal structures, site-directed mutagenesis, pH-dependence studies, or theoretical calculations^46^,^47^,^48^. Interestingly, glucose 1-oxidase/1-dehydrogenase has two conserved His residues, His505/His548 (AfGDH), His516/His559 (AnGOx), and His520/His563 (PaGOx). In addition, GDHs and GOxs have another conserved Glu residue (Glu399/Glu412/Glu416 for AfGDH/AnGOx/PaGOx, respectively) that forms a hydrogen bond with the conserved His. In the structure of AfGDH/LGC, the distances LGC (O1)–His505 (NE2), LGC (O1)–His548 (ND1), and LGC (C1)–FAD (N5) are 2.79, 2.57, and 2.99 Å, respectively (Fig. 5). The site-direct mutagenesis studies of His505 and His548, strongly supported that these residues are the catalytic pair, as were observed in GOx, because both mutants showed drastic decrease in the catalytic activity.

Although both His505 and His548 are residues that are potentially able to abstract a proton from the O1 of D-glucose, His548 is favored to be in a doubly protonated state, owing to the formation of a strong hydrogen bond with Glu399 interacting with Tyr337. Thus, His505 is likely to act as a general base that accepts the proton from the substrate because of its lower pKa, which is in consistent with that His505Ala almost lost its catalytic activity, whereas His548Ala remained slight GDH activity. A hydride transfer from the C1 of glucose to the N5 of the isoalloxazine ring then occurs, followed by the delocalization of electrons throughout the ring, resulting in D-glucono-delta-lactone and reduced FAD, as has been suggested for the substrate oxidation mechanism of GOxs (Fig. 5)^49^,^50^.

The application of fungus-derived FADGDHs as an ideal enzyme for blood glucose monitoring has been focused on their narrow substrate specificity, in particular their lack of activity toward maltose. Fungus-derived FADGDHs do not use oxygen as an electron acceptor and are accordingly insensitive to oxygen, their major advantage over GOx^51^. However, differences in substrate specificity between fungus-derived FADGDHs and GOx have been reported^11^,^12^,^13^,^14^,^15^,^16^,^19^. The second preferential substrate of fungus-derived FADGDHs is xylose, and their activity toward xylose is approximately 20% of that toward glucose, whereas GOxs do not react with xylose. Manufacturer manuals for glucose sensor strips clearly caution that strips employing fungus-derived FADGDHs should not be used with patients subjected to the xylose absorption test because of its inherent substrate specificity. AfGDH too shows activity toward xylose, approximately 20% of that toward glucose. The comparison of the substrate binding mode of fungal FADGDH by creating structural models based on homology analyses with GOxs, leaded to the conclusion that the residues recognizing glucose are conserved within fungal FADGDH and GOx, and no significant differences were observed 12. The structure of LGC complex provided the detailed information of substrate binding residues and an unique cavity of fungal FADGDH, which are inevitable and essential to understand the difference of substrate specificity between fungal FADGDH and GOx. As found in the AfGDH/LGC complex structure, the residues present within the hydrogen bond and van der Waals contact distances to LGC have been identified. Assuming that glucose can be replaced in the same position as LGC in the AfGDH/LGC complex structure, there are no marked differences between the residues of AfGDH recognizing glucose and those of GOx, as reported in the literature^21^. However, AfGDH lacks the residues near the sixth hydroxyl group of LGC/glucose (Fig. 4, Supplementary Figure S7). The GOx and glucose complex model has been reported previously for AnGOx^21^. In that report, it was suggested that GOxs recognize the sixth glucose hydroxyl group via interaction with Thr110 (AnGOx) or with Ser114 (PaGOx). Thr110 (AnGOx) and Ser114 (PaGOx) correspond to Ala96 of AfGDH, but the distance between the CB of Ala96 and the O6 of LGC is 4.98 Å (Supplementary Figure S7). The complex structure provided not only the information of residues with bound substrate, but also the differences of the size of the cavity for substrate binding from those of GOx. Figure 4 and Supplementary Figure S7 show that in the vicinity of the active site of both GOxs (AnGOx and PaGOx), Arg176/180 lies near the substrate-binding site, and it is located at a position forming hydrogen bonds with Asn217/221 and Gly108/112 and has van der Waals contact with Gln347/351. Unlike in GOxs, no residues from AfGDH belonging to the loop between B4 and H7 lie near the active site, and a cavity is present in the vicinity of the substrate-binding site. Therefore, the presence of this cavity in AfGDH may have a crucial role in the difference of the recognition of xylose compared with GOxs.

The absence of residues that recognize the sixth hydroxyl group of the glucose of AfGDH and the presence of significant cavity in the active site may account for this enzyme activity toward xylose. In other words, the introduction of a mutation and designing cavity in the vicinity of the region around the sixth hydroxyl group of LGC, as GOxs, would enable AfGDH to discriminate glucose from xylose, thereby showing no activity toward xylose.

**Figure 6:**
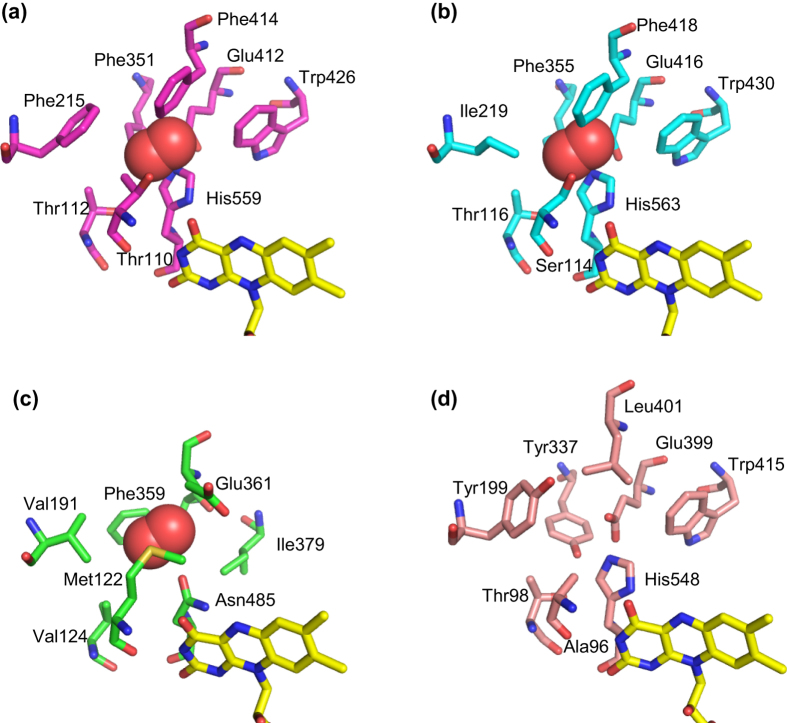
Comparison of residues responsible for the oxygen binding of AnGOx (1CF3), PaGOx (1GPE), ChOx (1MXT), and corresponding residues in AfGDH. (**a**) AnGOx (protein, magenta; FAD, yellow). (**b**) PaGOx (protein, cyan; FAD, yellow). (**c**) ChOx (protein, green; FAD, yellow). (**d**) AfGDH (protein, pink; FAD, yellow). Oxygen molecules are represented as red spheres.

A variety of oxidoreductases using FAD as a cofactor have been used for analytical purposes. However, electrochemical measurement using oxidases and electron mediators is potentially affected by the presence and variation of dissolved oxygen in analytes^51^. Remedying this inherent defect of oxidases, engineered oxidases have been reported to minimize oxidase activity and increase activity toward electron mediators by amino acid substitution, leading to the construction of dehydrogenases based on oxidases. Such engineering approaches have been reported for fructosyl amino acid oxidase^52^, fructosyl peptide oxidase^53^, cholesterol oxidase (ChOx)^54^, pyranose 2-oxidase^55^, and GOxs^56^,^57^. Engineering studies of GOxs have been performed on the basis of the structure of ChOx (1MXT), which binds an oxygen molecule in a predicted position^58^. ChOx belongs to the GMC oxidoreductase family. By superimposing AnGOx and PaGOx structures on ChOx with an oxygen molecule, we identified a region that possibly binds oxygen during the reaction of GOx. Mutagenesis studies of the residues of both ChOx^54^ and GOxs^56^,^57^ revealed mutant enzymes showing drastic decreases in oxidase activity but retaining dehydrogenase activity at similar or even higher levels compared with wild-type enzymes. Figure 6(a–c) show the regions and residues responsible for the oxygen binding of AnGOx, PaGOx, and ChOx. Figure 6(d) shows the corresponding region and residues of AfGDH. Among eight residues of GOx, four are identical and one is similar (unipolar). Interestingly, the remaining three residues, Ala96, Tyr199, and Tyr337, correspond to the crucial residues in GOxs and ChOx. Amino acid substitutions at Thr110 (AnGOx) or Ser114 (PaGOx) that correspond to Ala96, and/or at Phe351 (AnGOx) or Phe355 (PaGOx) that correspond to Tyr337 resulted in marked decreases in GOx activity, with some of the mutants showing the same or increased GDH activity. Similarly, amino acid substitutions at Val199 (ChOx), which corresponds to Tyr199, resulted in marked decreases in ChOx activity, with some of the mutants showing the same or increased cholesterol dehydrogenase activity. The elucidation of AfGDH structure revealed that these crucial residues in oxidases are not conserved in AfGDH, which is not able to use oxygen as the electron acceptor.

In conclusion, we report the 3D structure of fungus-derived glucose dehydrogenase using FAD as a cofactor. This is currently the most advanced and popular enzyme used in glucose sensor strips for SMBG. The structure of AfGDH consists of two major domains: an FAD-binding domain and a C-terminal domain. The overall structure was found to be similar to that of the fungal GOxs reported till date. The FAD molecule occupies a narrow channel. The ternary complex with reduced FAD and LGC revealed the residues that recognize the substrate. Although most of the residues are conserved with those reported in GOx, AfGDH lacks the residues near the sixth hydroxyl group, unlike those reported for GOxs. A large cavity in the vicinity of the active site is observed in AfGDH, whereas in GOxs, several residues occupy the corresponding cavity. These differences in the substrate recognition may account for the substrate specificity difference of AfGDH, which is active toward xylose. Residues predicted to associate with oxygen in GOxs are not conserved in AfGDH. These differences may account for the dehydrogenase characteristics of AfGDH.

The structural information reported here will contribute to the further engineering of fungus-derived FADGDH, such as by the improvement of substrate specificity and structural stabilization. These advances are expected to lead to increasingly reliable and economical biosensing technologies aimed at diabetes management.

## Methods

### Recombinant production of AfGDH

We prepared recombinant nonglycosylated FADGDH derived from *A. flavus* using *Escherichia coli* as the host microorganism. The expression and purification of *A. flavus* glucose dehydrogenase (NCBI reference sequence: XP_002372599.1; AfGDH) have been reported previously^20^. Briefly, the structural gene of AfGDH was synthesized using GenScript Inc. (Piscataway, NJ, USA), with codons optimized for expression in *E. coli*. After the removal of the sequence encoding the secretion signal peptide (amino acids 1 to 22), which were predicted using SignalP 3.0^59^,^60^, the amino acid sequence of AfGDH corresponding to the 24–593 region was expressed using plasmid (pET30C) encoding the *AfGDH* gene in *E. coli* Origami2 (DE3) cells and purified by anion exchange chromatography using a Resource Q column (GE Healthcare Life Sciences, Uppsala, Sweden). The purified protein solution was dialyzed overnight against 10 mM potassium phosphate buffer (pH 6.5).

### Site-directed mutagenesis studies

Site-directed mutagenesis was performed by overlap extension PCR using mutagenic primers and the AfGDH structural gene as template. Mutant PCR products were digested with the *Nde*I and *Hin*dIII and inserted into the multicloning site of the expression vector pET-30C.

### Enzyme assay

The enzymatic activity of purified wild-type AfGDH and its mutants was determined as follows. Each 20 μL sample was mixed with 160 μL reaction buffer [10 mM potassium phosphate buffer (pH 6.5), 0.06 mM 2,6-dichlorophenol indophenol (DCIP), and 0.6 mM phenazine methosulfate (PMS)]. The reaction was initiated by injecting 20 μL of 2M D-glucose (200 mM final concentration) and the rate of the reaction was determined by monitoring the decrease in absorbance of DCIP at 600 nm at 25 °C. The protein concentrations were measured using the DC Protein Assay Kit (Bio-Rad; Hercules, CA, USA) with bovine serum albumin as standard.

### Crystallization

The purified protein solution was concentrated to 11.5 mg/mL for crystallization using an Amicon Ultra-0.5 mL centrifugal filter (NMWL: 30 kDa) (Merck Millipore, Billerica, MA, USA). The initial screening was performed by the sitting-drop vapor-diffusion method with VIOLAMO 96 well-plates (AS ONE Corp., Osaka, Japan) using the mosquito system (TTP LabTech, Hertfordshire, UK) at 293 K. Some crystals appeared in several reservoir solutions using Crystal Screen kits 1 & 2; Index Screen I & II; PEG/Ion 1 & 2; Salt RX Screen (Hampton Research Corp., CA, USA); and Wizard I, II, III, & IV (Emerald BioSystems, Inc., WA, USA). After optimization of the crystallization condition, well-diffracting crystals were obtained in a droplet containing 1.0 μL of protein solution (11.5 mg/mL in 10 mM of potassium phosphate buffer, pH 6.5) and 1.0 μL of reservoir solution (0.1 M of BisTris, pH 6.5, 22–25% PEG3350) against 80 μL of the reservoir solution by the sitting-drop method using a 96-well plate (Corning Inc., NY, USA) at 293 K.

### Data collection and structure determination

A single crystal mounted in a cryoloop was directly flash-cooled in a stream of evaporating nitrogen. X-ray diffraction data were collected using an ADSC Quantum 315r detector on the BL5A in the Photon Factory (Tsukuba, Japan). The data were processed using an HKL2000 system^61^ and the CCP4 program suite (Collaborative Computational Profect 4, 1994)^62^. For obtaining a ligand-complexed structure, a single crystal was soaked in 15% (w/v) LGC in reservoir solution for 30 s and flash-cooled. The data collection statistics are summarized in Table 1.

Initial phase determination and model building of AfGDH were performed by molecular replacement with the program AutoMR and Autobuild in the Phenix system^63^ using the structure of AnGOx (PDB ID: 1CF3^21^) as a probe model. Further model building and refinement were performed using the programs Coot^64^ and Refmac5^65^, respectively. Water molecules were introduced into the peaks of the (*Fo*-*Fc*) electron density map (above 3.0 σ) with the range of a hydrogen bond (2.5–3.5 Å) using Coot. A bound FAD in the active site was refined as a reduced form (FADH_2_) considering the observed electron density map of the bending isoalloxazine ring of FAD. The structure of AfGDH was refined at 1.78 Å. The complex structure with LGC was determined by isomorphous replacement using the structure of AfGDH and was refined at 1.57 Å. The number of residues in the most favored regions in a Ramachandran plot^66^ was determined using Coot. Refinement statistics are presented in Table 1. Figures 1,3, 4, 5, 6, and Supplementary Figures S1, S2, S5–S7 were constructed using the program PyMol (Schrödinger, LLC., New York, USA).

## Additional Information

**Accession codes:** The atomic coordinates and structure factors of AfGDH and AfGDH/D-glucono-1,5-lactone have been deposited in the Protein Data Bank under accession codes 4YNT and 4YNU, respectively.

**How to cite this article**: Yoshida, H. *et al.* Structural analysis of fungus-derived FAD glucose dehydrogenase. *Sci. Rep.* **5**, 13498; doi: 10.1038/srep13498 (2015).

## Supplementary Material

Supplementary Information

## Figures and Tables

**Table 1:** Data collection and refinement statistics.

	AfGDH	AfGDH/LGC
Data collection		
Beamline	PF BL5A	PF BL5A
Temperature (K)	100	100
Wavelength (Å)	1.0	1.0
Resolution range (Å)	50.0–1.77	50.0–1.57
	(1.80–1.77)	(1.60–1.57)
No. of measured refs.	143,309	242,808
No. of unique refs.	45,130 (2,102)	65,992 (3,221)
Redundancy	3.2 (2.4)	3.7 (3.0)
Completeness (%)	99.0 (93.4)	99.9 (97.6)
Mean *I*_*o*_/σ(*I*_*o*_)	15.2 (2.4)	10.6 (2.2)
*R*_*merge*_ (%)	7.1 (46.2)	5.9 (48.0)
Space group	*P*2_1_	*P*2_1_
Unit cell parameters	*a* = 49.91	*a* = 49.66
*a, b, c* (Å)	*b* = 64.36	*b* = 64.88
β (°)	*c* = 76.17	*c* = 75.89
	β = 101.43	β = 100.46
Refinement		
Resolution range (Å)	48.75–1.78	39.02–1.57
	(1.82–1.78)	(1.61–1.57)
No. of refs.	42,833 (2,799)	62,625 (4,477)
Completeness (%)	98.5 (87.8)	99.7 (96.6)
*R*_*factor*_ (%)	15.6 (23.1)	14.2 (22.2)
*R*_*free*_ (%)	19.9 (34.5)	16.4 (26.9)
RMSD bond lengths (Å)	0.006	0.007
RMSD bond angles (°)	1.0	1.1
Ramachandran plot		
Preferred region (%)	95.6	96.9
Allowed region (%)	4.1	2.7
*B*-factor (Å^2^)		
Protein	23.6	20.3
Cofactor reduced FAD	16.9	14.4
Ligand binding to the catalytic site		22.0 (LGC)
Ligand binding to the protein surface		49.5 (LGC)
Water	30.5	28.1
PDB code	4YNT	4YNU

Values in parentheses are of the high-resolution bin. *R*_merge_ = Σ_*h*_ Σ_*i*_ [|*I*_*i*_(*h*) – <*I*(*h*)>|/Σ_*h*_ Σ_*i*_ *I*_*i*_(*h*)], where *I*_*i*_ is the _*i*_th measurement and <*I*(*h*)> is the weighted mean of all measurements of *I*(*h*).
