# Supplementary material for: Structural analysis of fungus-derived FAD glucose dehydrogenase
Source: Sci Rep. 2015 Aug 27;5:13498. doi: 10.1038/srep13498 (PMC4642536; doi:10.1038/srep13498)
Supplement: Supplementary Information [file srep13498-s1.pdf]

Structural analysis of fungus-derived FAD glucose dehydrogenase

Hiromi Yoshida[1], Genki Sakai[2], Kazushige Mori[3], Katsuhiro Kojima[3], Shigehiro Kamitori[1], and Koji Sode[2,3,*]

[1] Life Science Research Center and Faculty of Medicine, 1750-1, Ikenobe, Miki-cho, Kita-gun, Kagawa University, Kagawa 761-0793, Japan

[2] Department of Biotechnology and Life Science, Graduate School of Engineering, Tokyo University of Agriculture and Technology, 2-24-16, Nakamachi, Koganei, Tokyo 184-8588, Japan

[3] Ultizyme International Ltd., 1-13-16, Minami, Meguro, Tokyo 152-0013, Japan

[*] Corresponding author

Correspondence to: sode@cc.tuat.ac.jp

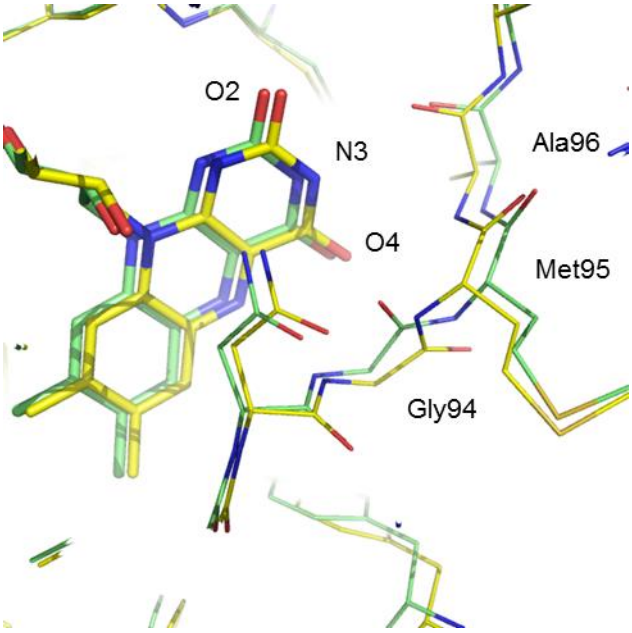

Supplementary Figure S1

Reordered main-chain of AfGDH with bound gluconolactone.

The structures around isoalloxiazine ring of FAD in AfGDH (yellow) and AfGDH/LGC (green) were shown in stick and line model. The bound LGC is not represented.

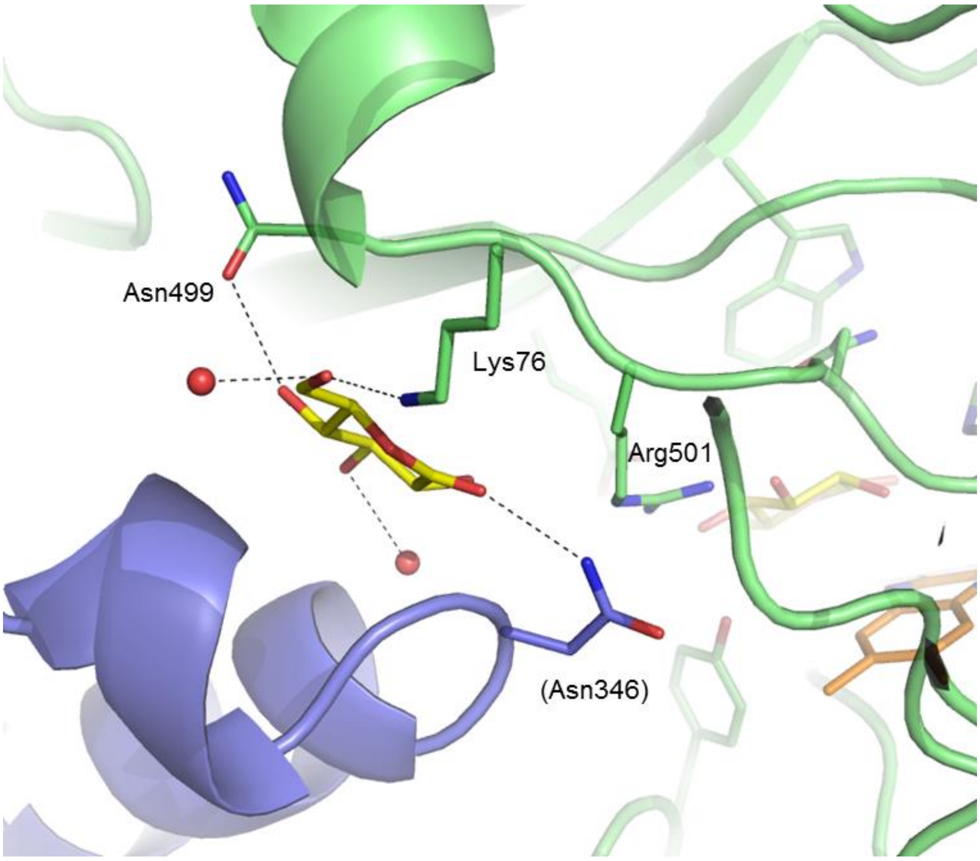

Supplementary Figure S2

A bound LGC on molecular surface.

A bound LGC was located at inter-molecular surface in the crystal, forming hydrogen bonds with Lys76, Asn499, (Asn346), and two water molecules. The label with parenthesis belong to the neighbouring molecule (light purple) in molecular packing . Water molecules interacting with LGC are represented as red spheres.

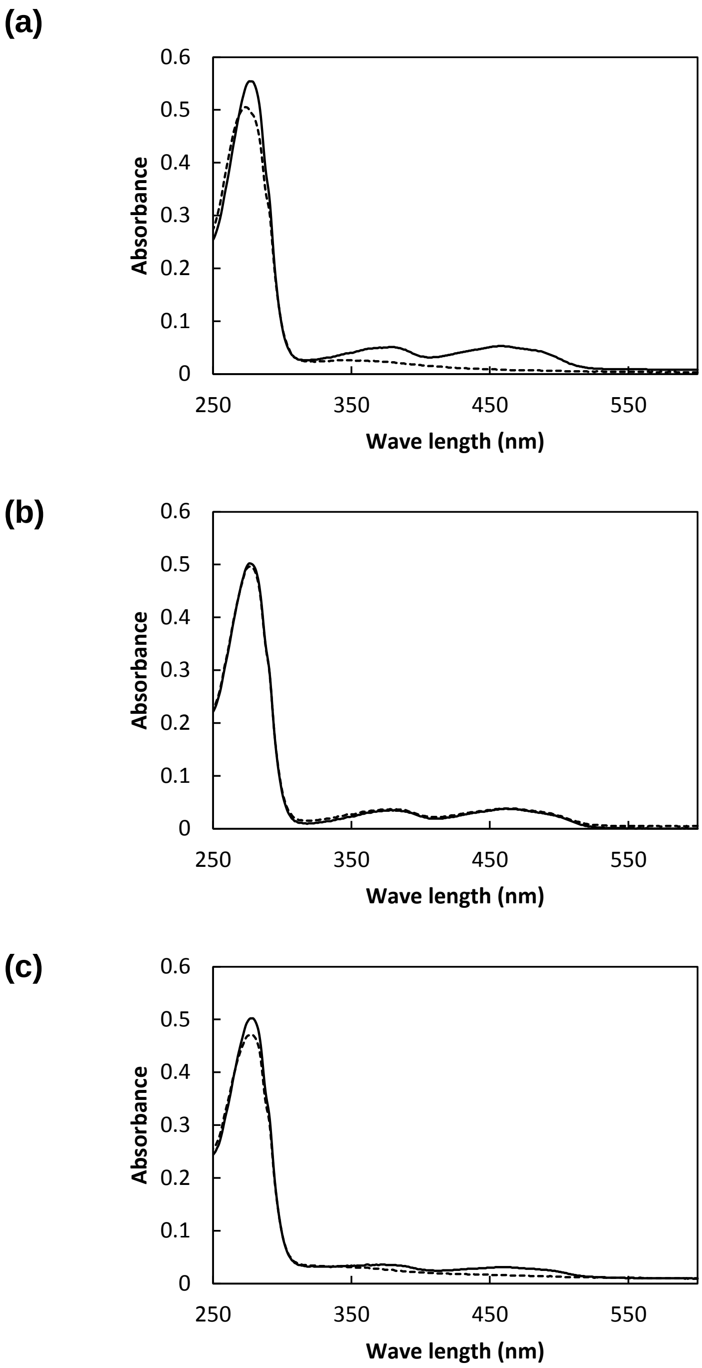

Supplimentary Figure S3

UV-VIS spectra analysis for reduction of AfGDHs by glucose

(a) Wild-type (b) His505Ala (c) His548Ala

The solid line represents the absorption spectrum of AfGDH in the absence of glucose, and the dotted line shows the absorption spectrum after incubation with 20 mM glucose for 1 min. Each spectra was measured for 300μg/mL enzyme in 10mM potassium phosphate buffer pH6.5.

(a)

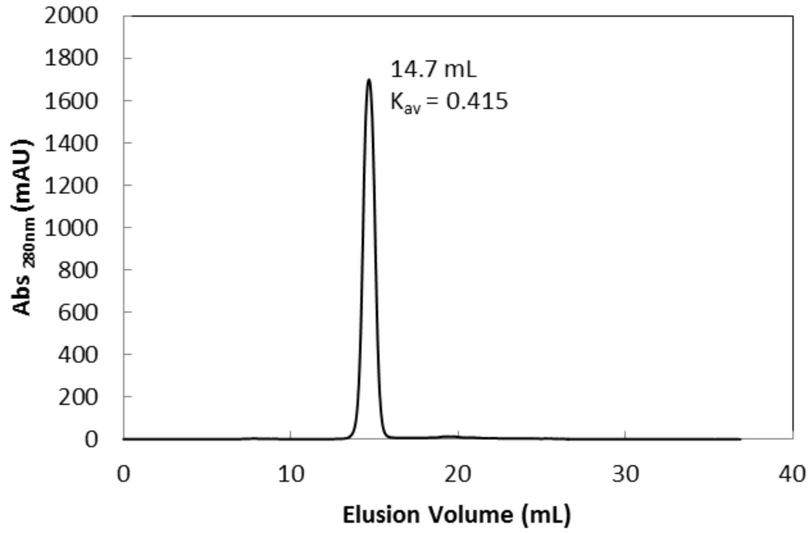

(b)

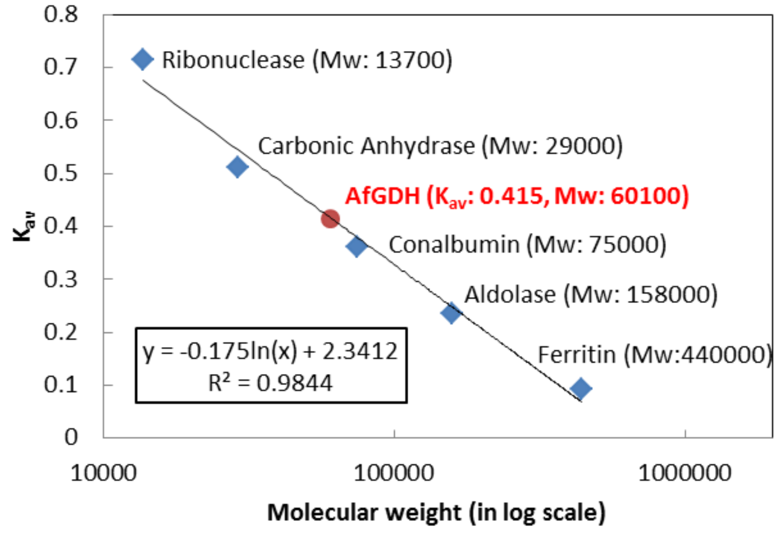

Supplementary Figure S4

Molecular mass estimation of AfGDH using size exclusion chromatography

(a) Chromatogram of purified AfGDH on Superdex 200 10/300 GL column.

(b) Calibration curve

Purified AfGDH and standard proteins were applied to the Superdex 200 10/300 GL column (GE Healthcare Life Sciences, Uppsala, Sweden) equilbrated with 10mM potassium phosphate buffer pH6.5. The flow rate was 0.5mL/min.

K_{av}: partition coefficient

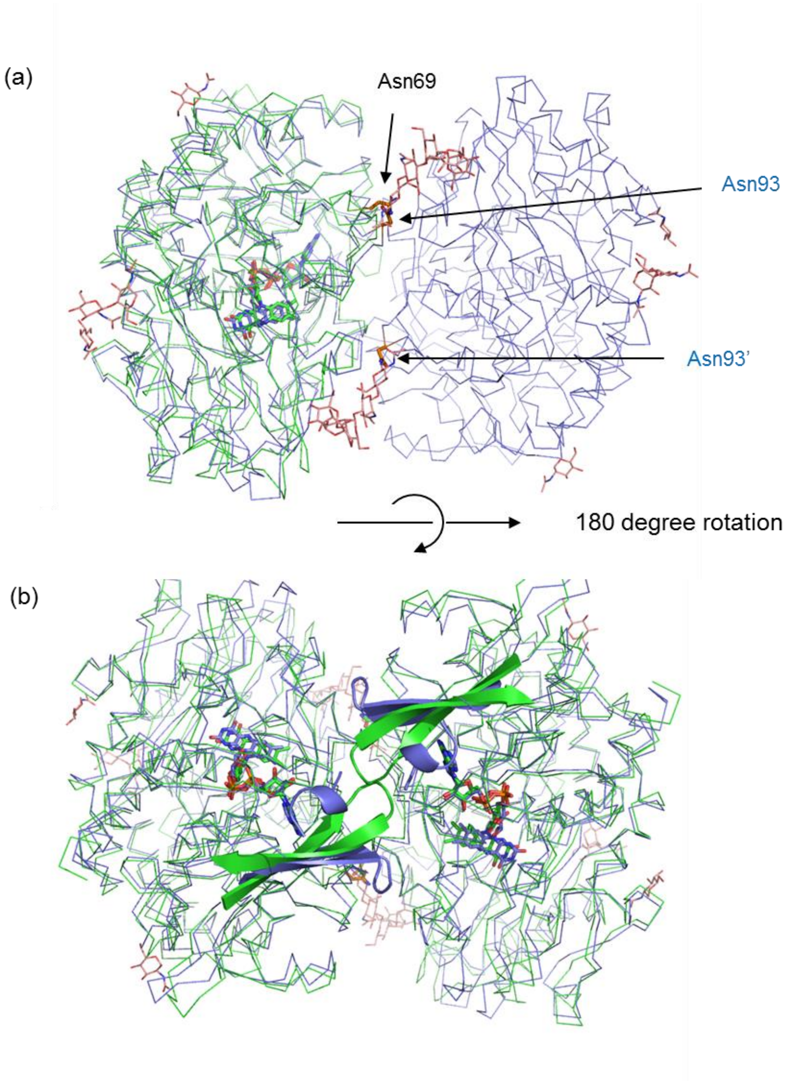

Supplementary Figure S5

Comparison of dimeric structure of glucose oxidase PaGOx (1GPE, purple) and monomer structure of AfGDH/LGC (green)

The intriguing glycosylation site of PaGOx (Asn93) and a potential glycosylation site of AfGDH (Asn69) are shown in orange stick model. The bound LGCs are not shown.

(a) Observed carbohydrates on PaGOx are presented in pink stick model and the extended carbohydrates attached to Asn93 reach to the other molecule each other for stabilizing dimer conformation[21]. (b) An additional AfGDH was superimposed onto the dimer form of PaGOx. A purple colored β-turn and N-terminal region including a short α-helix of PaGOx are located at dimer interface. The green colored β-turn between B10 and B11 of AfGDH coresponding to the above β-turn of PaGOx is directed toward opposite site (to the center of dimer form of PaGOx), owing to a lack of corresponding N-terminal region. Such a β-turn caused stearic hindrance at dimer interface if AfGDH forms dimer as has been in PaGOx.

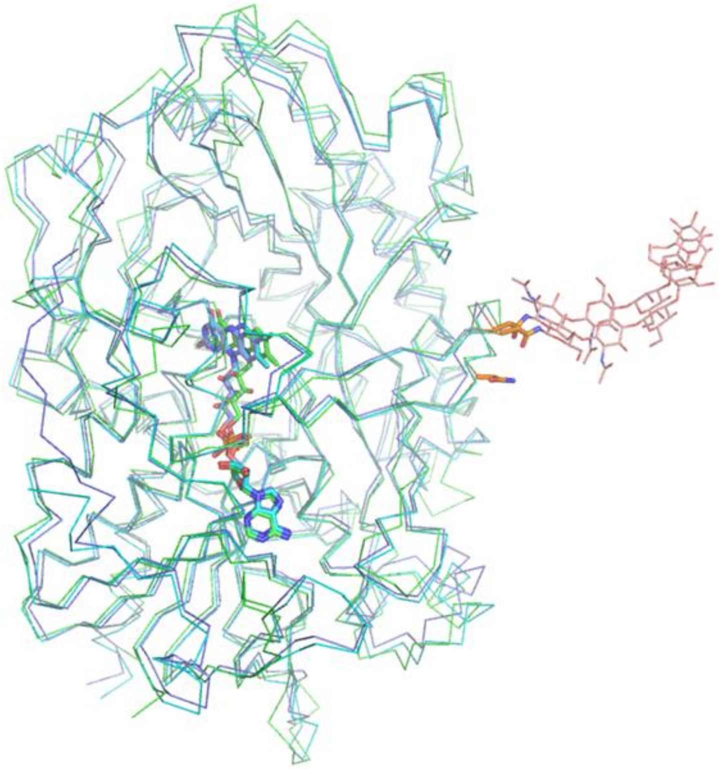

Supplementary Figure S6

Superimposed overall structure of glucose oxidases (AnGOx (1CF3, cyan) and PaGOx (1GPE, purple)) on AfGDH/LGC (green)

The glycosylation sites of AnGOx (Asn89) and PaGOx (Asn93) are shown in orange stick model and the observed extended carbohydrates are shown in pink line[21]. An potential glycosylation site of AfGDH (Asn69) is colored in orange. The bound LGCs are not shown.

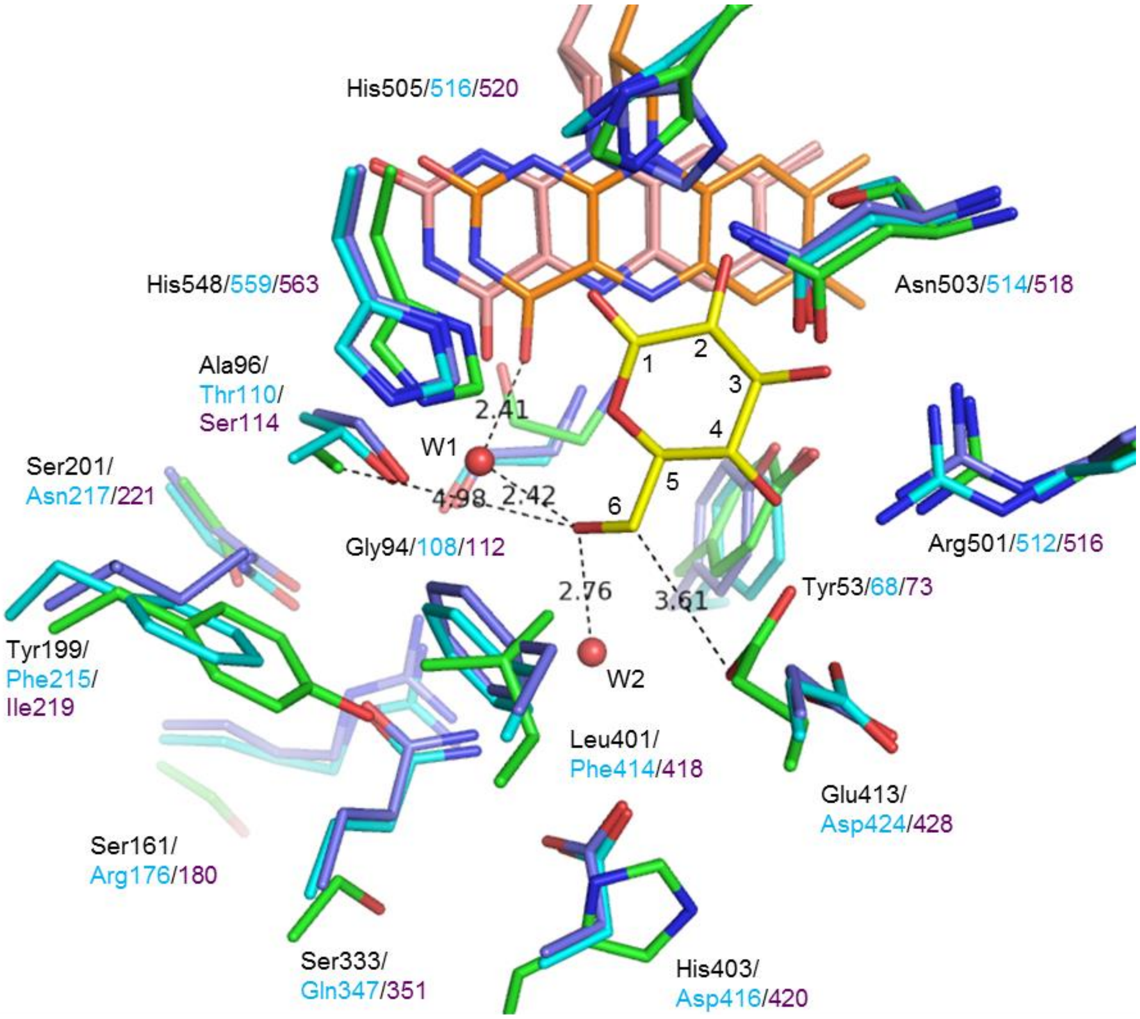

Supplementary Figure S7

The 6th hydroxyl group recognition of gluconolactone

Comparison of active site structures among AfGDH/LGC, AnGOx (1CF3), and PaGOx (1GPE).

The active site structures of AnGOx (protein: cyan; FAD: pink) and PaGOx (protein: purple; FAD: pink) were superimposed onto that of AfGDH/LGC (protein: green; FAD: orange; LGC: yellow) . The label of residues belong to AfGDH and followed by AnGOx and PaGOx. The water molecules interacted with LGC are represented in red spheres. Dotted lines show the distances between selected atoms.

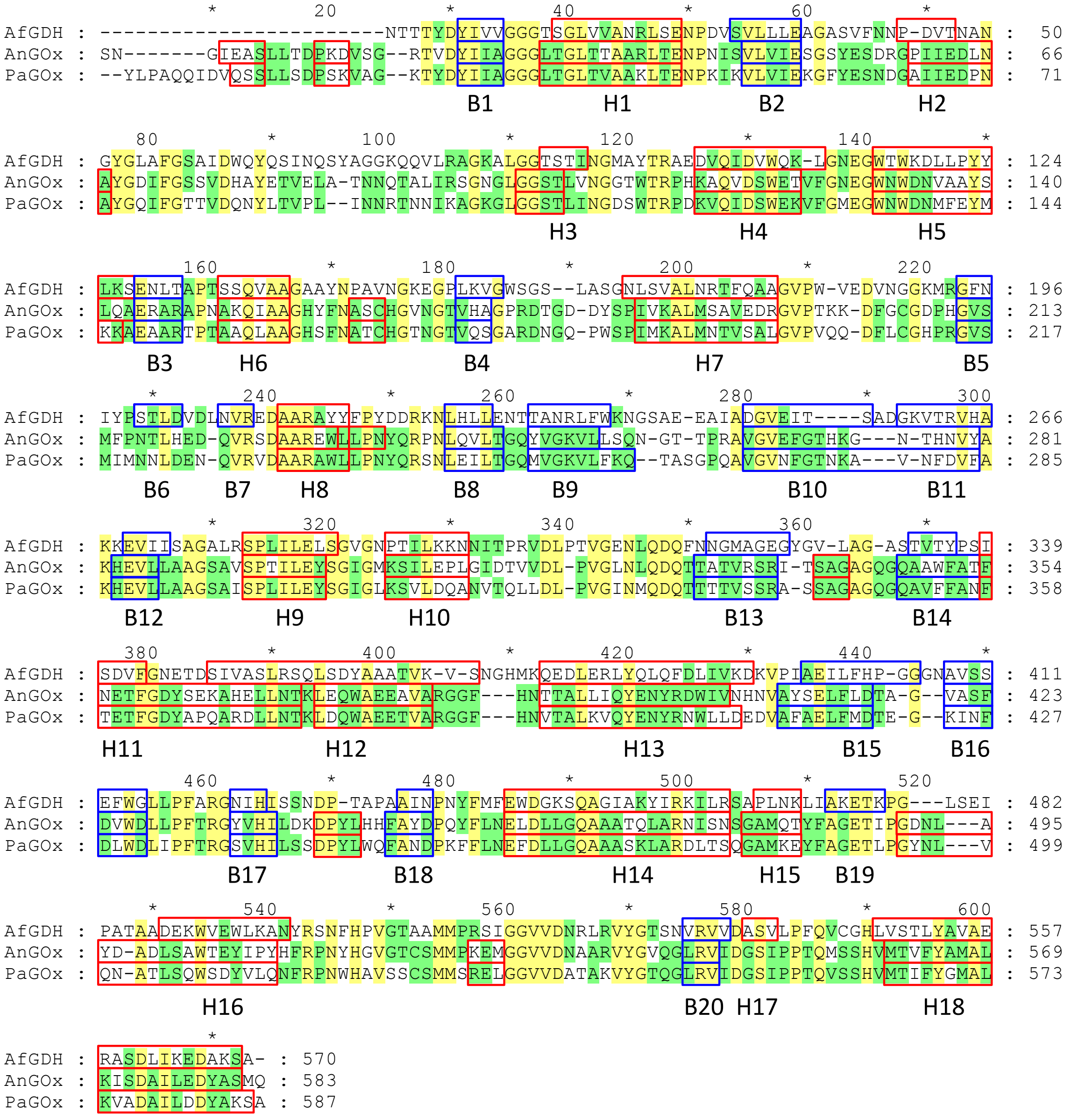

Supplementary Figure S8

Sequence alignment of AfGDH and GOxs.

FADGDH from *A. flavus* (AfGDH) is aligned with glucose oxidases from *Aspergillus niger* (AnGOx; 1CF3) and *Penicillium amagasakiense* (PaGOx; 1GPE).

Red and blue boxes indicate α-helix and β-strand, respectively. The number with secondary structure region belongs to the structure of AfGDH.

Supplementary Table S1. Selected hydrogen bond and van der Waals contact distance to reduced FAD (< 3.3 Å).

Cofactor	AfGDH	Distance (Å)	AfGDH/LGC	Distance (Å)
FDA N5F	Gly94 N	3.15	Gly94 N	3.21
FDA N5F			LGC C1	2.99
FDA N5F			LGC O1	3.07
FDA N5F			LGC O2	3.05
FDA O4F	Met95 N	3.14	Gly94 N	3.21
FDA O4F	Ala96 N	2.96	Gly94 O	2.93
FDA O4F			His548 ND1	3.27
FDA O4F			HOH 231	2.41
FDA N3F	Ala96 O	2.89	His548 ND1	3.21
FDA O2F	Ala96 O	3.20		
FDA O2F	Val550 N	2.94	Val550 N	2.82
FDA O2F	HOH 6	2.91	HOH 12	3.24
FDA O2’	Asn93 ND2	3.08	Asn93 ND2	2.99
FDA O3’	HOH 69	2.98	HOH 20	3.00
FDA O4’	Thr15 OG1	2.77	Thr15 OG1	2.76
FDA O5’	Thr15 OG1	3.17	Thr15 OG1	3.19
FDA O1P	Ser16 N	3.03	Ser16 N	3.00
FDA O1P	Ser16 OG	2.80	Ser16 OG	2.81
FDA O1P	HOH 1	3.01	HOH 7	2.95
FDA O2P	Ala538 N	3.16	Ala538 N	3.12
FDA O2P	HOH 31	2.73	HOH 1	2.74
FDA O1A	Thr89 OG1	2.64	Thr89 OG1	2.65
FDA O1A	HOH 36	2.62	HOH 4	2.79
FDA O1A	HOH 57	2.82	HOH 27	2.78
FDA O2A	Thr15 N	3.17	Thr15 N	3.15
FDA O2A	Thr15 OG1	2.66	Thr15 OG1	2.76
FDA O2A	Thr89 N	2.90	Thr89 N	2.88
FDA O5B	HOH 1	3.25	HOH 7	3.26
FDA O3B	Glu36 OE1	2.68	Glu36 OE1	2.68
FDA O3B	Glu36 OE2	3.24	Glu36 OE2	3.22
FDA O3B	Ala85 O	3.02	Ala85 O	3.03
FDA O2B	Glu36 OE2	2.73	Glu36 OE2	2.74
FDA O2B	Trp63 NE1	3.17	Trp63 NE1	3.07
FDA O2B	Gly83 O	3.20	Gly83 O	3.24
FDA N1A	Ala235 N	2.96	Ala235 N	2.86
FDA N3A	Ala37 N	3.25	Ala37 N	3.22
FDA N6A	Ala235 O	2.88	Ala235 O	2.87

Supplementary Table S2. Selected hydrogen bond and van der Waals contact distances to bound LGC (< 3.3 Å).

Ligand	AfGDH	Distance (Å)	AfGDH/LGC	Distance (Å)
LGC C1			FDA N5F	2.99
LGC O1			His548 ND1	2.57
LGC O1			FDA C4F	2.85
LGC O1			FDA N5F	3.07
LGC O2			Asn503 O	2.66
LGC O2			His505 NE2	2.89
LGC O2			FDA N5F	3.05
LGC O3			Arg501 NH1	2.83
LGC O3			Asn503 OD1	2.78
LGC O4			Tyr53 OH	2.71
LGC O4			Glu413 OE1	2.60
LGC O4			Arg501 NH2	2.89
LGC O6			HOH 231	2.42
LGC O6			HOH 362	2.76
